# Evaluation of Use of Shorter Radiation Regimens for Breast and Prostate Cancer in the US, 2015-2017

**DOI:** 10.1001/jamanetworkopen.2020.10519

**Published:** 2020-07-16

**Authors:** Erin F. Gillespie, Kathryn R. Tringale, Peter B. Bach, Justin E. Bekelman

**Affiliations:** 1 Department of Radiation Oncology, Memorial Sloan Kettering Cancer Center, New York, New York; 2 Center for Health Policy and Outcomes, Memorial Sloan Kettering Cancer Center, New York, New York; 3 Penn Center for Cancer Care Innovation, Abramson Cancer Center, University of Pennsylvania, Philadelphia

## Abstract

This cross-sectional study examines the usage rates and trends of shorter vs longer radiation regimens for breast cancer and prostate cancer treatments in the US.

## Introduction

For breast and prostate cancer, shorter radiation treatment regimens lasting 3 to 5 weeks are evidence-based practices that are similarly effective and safe, and substantially less costly for payers and patients, compared with extended regimens lasting 6 to 9 weeks.^1^ National guidelines endorsed shorter radiation regimens for breast cancer in 2011 and for prostate cancer in 2018.^2,3^ In July 2019, the Centers for Medicare & Medicaid Services proposed a mandatory episode-based payment model for radiation services, partly motivated by an interest in accelerating uptake of shorter radiation regimens, and publicly released Medicare data on US radiation episodes during the period from 2015 to 2017.^4^ During this period prior to guideline endorsement of shorter regimens for prostate cancer, we hypothesized that growth in uptake of shorter regimens would be greater in breast cancer than in prostate cancer.

## Methods

The data set contains radiation episodes covering 84% of Medicare beneficiaries.^4^ In this cross-sectional study, we included beneficiaries with breast and prostate cancer treated with external radiation (conventional, intensity-modulated, or proton radiotherapy). We classified episodes into 2 groups: shorter regimens (11 to 20 daily treatments for breast cancer or 11 to 30 for prostate cancer) and extended regimens (>20 or >30 daily treatments, respectively). The study was approved as exempt for the need for informed consent by the Memorial Sloan Kettering Cancer Center institutional review board because publicly available anonymized data were used. The study followed the Strengthening the Reporting of Observational Studies in Epidemiology (STROBE) reporting guidelines.

We calculated compound annual growth rates and used multivariable linear regression to compare rates of change in the use of shorter regimens between breast and prostate cancer. We compared radiation-related spending for shorter vs extended regimens from the amount reimbursed by Medicare for facility and professional services over the 90-day episode, adjusted for inflation to 2017. Statistical significance was set at 2-sided *P* <* .*025, applying a Bonferroni correction for 2 main analyses. Data analysis was performed from September to December 2019 using SAS Enterprise Guide statistical software version 9.1 (SAS Institute).

## Results

From 2015 to 2017, among 85 570 radiation episodes for women with breast cancer aged 65 to 74 years (67%), 75 to 84 years (28%), and 85 years or older (19%), shorter radiation regimens increased from 33.1% (95% CI, 32.5%-33.6%) to 42.4% (95% CI, 41.9%-43.0%) (*P* < .001) at a compound annual growth rate of 13.2%. Among 71 720 episodes for men with prostate cancer aged 65 to 74 years (63%), 75 to 84 years (33%), and 85 years or older (4%), shorter regimens increased from 13.4% (95% CI, 13.0%-13.9%) to 16.7% (95% CI, 16.2%-17.2%) (*P* < .001) at a compound annual growth rate of 11.6% (Figure). Rates of change in use of shorter regimens did not differ significantly between the 2 cancers (compound annual growth rate, 13.2% vs 11.6%; difference, 1.6%).

**Figure.**
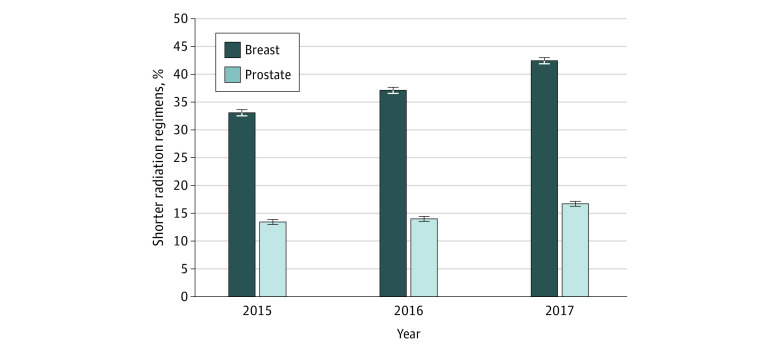
Proportion of Shorter Radiation Regimens for Breast and Prostate Cancer, 2015 to 2017 Bars denote proportion of shorter radiation regimens by each year chronologically and stratified by cancer type, with overlying lines to demonstrate the longitudinal trend. Error bars denote 95% confidence intervals.

Mean 90-day radiation-related spending was 33% lower for beneficiaries with breast cancer treated with shorter compared with extended regimens ($9204 [95% CI, $9179-$9229] vs $13 733 [95% CI, $13 707-$13 759]; difference = $4529; *P* < .001) and 34% lower for prostate cancer ($18 709 [95% CI, $18 580-$18 839] vs $28 262 [95% CI, $28 217-$28 307]; difference = $9553; *P* < .001) (Table).

**Table.** Spending on Radiation Regimens for Medicare Beneficiaries with Breast and Prostate Cancer, 2015 to 2017

Radiation regimen (No. of fractions)	Episodes, No. (%) [95% CI]	Cost, mean (95% CI), $	*P* value
Breast			
Shorter (11-20)	32 178 (37.6) [37.3-37.9]	9204 (9179-9228)	<.001
Extended (>20)	53 362 (62.4) [62.1-62.7]	13 733 (13 707-13 759)	
Prostate			
Shorter (11-30)	10 549 (14.7) [14.5-15.0]	18 709 (18 580-18 839)	<.001
Extended (>30)	61 021 (85.3) [85.0-85.5]	28 262 (28 217-28 307)	

## Discussion

Among Medicare beneficiaries receiving radiation treatment between 2015 and 2017, the rate of uptake of shorter radiation regimens was modest and did not differ meaningfully between breast and prostate cancer. We also found that shorter radiation regimens for prostate cancer, like breast cancer, reduce radiation-related spending by approximately one-third. During the study period, guidelines had endorsed shorter regimens for breast cancer but not prostate cancer; comparable uptake underscores the challenge of implementing less costly evidence-based practices in cancer care.^5^

Today, accelerating uptake of shorter radiation regimens is an urgent priority to enhance evidence-based, patient-centered cancer care. Development, testing, and scaling of strategies to achieve this goal, such as default options, audit and feedback and patient engagement, is warranted.^6^

This study has limitations. Because the Centers for Medicare & Medicaid Services data set is missing cancer stage and radiation field, these results underestimate the absolute proportion of beneficiaries receiving shorter regimens. However, absent evidence of secular changes in cancer stage or treatment patterns, rate of uptake of shorter regimens is unlikely to be underestimated.
